# NetworkPainter: dynamic intracellular pathway animation in Cytobank

**DOI:** 10.1186/s12859-015-0602-4

**Published:** 2015-05-25

**Authors:** Jonathan R Karr, Harendra Guturu, Edward Y Chen, Stuart L Blair, Jonathan M Irish, Nikesh Kotecha, Markus W Covert

**Affiliations:** 1 Graduate Program in Biophysics, Stanford University, 443 Via Ortega, MC 4245, Stanford, CA 94305 USA; 2 Department of Electrical Engineering, Stanford University, 279 Campus Drive West, MC 5329, Stanford, CA 94305 USA; 3 Department of Bioengineering, Stanford University, 443 Via Ortega, MC 4245, Stanford, CA 94305 USA; 4 Department of Medicine, Stanford University, 269 Campus Drive West, MC 5175, Stanford, CA 94305 USA; 5 Department of Microbiology & Immunology, Stanford University, 269 Campus Drive West, MC 5175, Stanford, CA 94305 USA; 6 Graduate Program in Biomedical Informatics, Stanford University, 269 Campus Drive West, MC 5175, Stanford, CA 94305 USA; 7 Cytobank Inc, 821 West El Camino Real, Mountain View, CA 94040 USA; 8 Department of Genetics & Genomic Sciences, Mount Sinai School of Medicine, One Gustave L Levy Place, New York, NY 10029 USA; 9 Department of Cancer Biology, Vanderbilt University, 740B Preston Building, 2220 Pierce Avenue, Nashville, TN 37232 USA

**Keywords:** Visualization, Animation, Network, Systems biology, Cytometry

## Abstract

**Background:**

High-throughput technologies such as flow and mass cytometry have the potential to illuminate cellular networks. However, analyzing the data produced by these technologies is challenging. Visualization is needed to help researchers explore this data.

**Results:**

We developed a web-based software program, NetworkPainter, to enable researchers to analyze dynamic cytometry data in the context of pathway diagrams. NetworkPainter provides researchers a graphical interface to draw and “paint” pathway diagrams with experimental data, producing animated diagrams which display the activity of each network node at each time point.

**Conclusion:**

NetworkPainter enables researchers to more fully explore multi-parameter, dynamical cytometry data.

**Electronic supplementary material:**

The online version of this article (doi:10.1186/s12859-015-0602-4) contains supplementary material, which is available to authorized users.

## Background

Cellular signaling is enormously complex, arising from dynamic interactions among thousands of molecules. Understanding signaling at the molecular level therefore requires comprehensive, dynamic measurements of individual cells [1]. Flow and mass cytometry are two of the most informative technologies, capable of measuring multiple properties of thousands of individual cells per second [2]. Fluorescence-based flow cytometry enables up to 12 simultaneous measurements [3]. Mass cytometry promises as many as 100 [4]. Flow cytometry has provided crucial information about DNA copy number [5], protein expression [6], and phenotypes [7]. Mass cytometry has been used to study leukemia [8], hematopoiesis [9], and immune signaling [10].

Recently, Kotecha and colleagues developed Cytobank, a web-based platform for storing, exploring, and sharing cytometry data [http://www.cytobank.org; [11]. Cytobank stores primary flow and mass cytometry data and provides annotation, gating, and visualization tools. Cytobank also enables researchers to easily share data and analysis with collaborators. Cytobank’s vertical integration enables researchers to drill down from publication quality figures to the underlying analysis, processing, filtering, and ultimately to the raw data, making data analysis fully transparent and reproducible. Additional tools are needed to help researchers analyze dynamic data.

Visualization is an effective technique for investigating complex data [12-14]. Flow cytometry researchers commonly use several graphical analysis software programs and packages including FlowJo [http://www.flowjo.com], flowViz [15], flowCore [16], iFlow [17], and SPICE [18] to browse and analyze cytometry data. Recently, researchers have developed several new techniques for visually analyzing mass cytometry data. Qui et al. developed spanning-tree progression analysis of density-normalized events (SPADE) to cluster and visualize measurements across multiple cell types [19]. Amir et al. developed viSNE to analyze mass cytometry data using visuals similar to scatter plots [20].

In addition, several tools are available for visualizing temporal data in the context of the underlying molecular network. Secrier and Schneider have extensively reviewed these dynamical data visualization tools [14]. These tools use three main strategies to visualize temporal data on top of networks. First, several tools including TVNViewer [21] and VisANT [22] can depict temporal dynamics using multiple vertically stacked or spatially tiled layers corresponding to different time points. Each layer displays the same network structure and colors and/or boldens the observed nodes and edges according to their observed activity. Second, several tools including the Cytoscape [23] plugins MODAM [24], MultiColoredNodes [25], SpotXplore [26], and VistaClara [27], and VANTED [28] place small sub-plots next to each observed node and edge to indicate its observed temporal dynamics. Third, several tools including BioTapestry [29], DynNetwork [http://code.google.com/p/dynnetwork], and the Pathway Tools Cellular Overview [30] animate network diagrams by coloring or boldening each observed node and edge at each time point. To optimally display large networks and high-dimensional data, these existing network visualization tools depict networks using streamlined node-link or circuit diagrams.

However, cell signaling researchers often prefer classical textbook-style pathway diagrams for medium size networks and multi-parameter data. Unfortunately, none of the textbook-style pathway drawing tools such as ChemBioDraw [http://www.cambridgesoft.com/software/overview.aspx] and Cell Illustrator [31] are capable of displaying temporal dynamics. These software programs are also proprietary and cannot be integrated or embedded within other software or databases. CellDesigner, which provides a graphical style intermediate between the streamlined node-link and classical styles, also cannot display dynamics directly on top of a network [32].

We developed a web-based software program, NetworkPainter, to enable cell signaling researchers to visualize and communicate multi-parameter data in the context of classical textbook-style pathway diagrams. NetworkPainter enables researchers to draw and “paint” classical pathway diagrams with data, creating animated diagrams. Optionally, NetworkPainter also displays heatmaps next to each observed node to indicate its underlying activity distribution across different experimental conditions or patients. Furthermore, we have integrated NetworkPainter with Cytobank to encourage experimentalists to try this visualization without any complicated importing or exporting. In addition, we developed a standalone version of NetworkPainter available at http://covert.stanford.edu/networkpainter which is capable of visualizing any dynamic data. Both versions also enable researchers to share diagrams with collaborators.

NetworkPainter was motivated by the unique needs of flow- and mass-cytometry researchers to visualize multi-parameter data. NetworkPainter fills a dynamical data visualization need unmet both by existing network visualization software programs which are optimized for larger networks and by existing classical pathway drawing tools which are not capable of displaying temporal dynamics. NetworkPainter combines the capabilities of classical pathway drawing tools with the capabilities of network visualization tools to visualize dynamics through animation and multiple sub-plots. We believe that NetworkPainter can help scientists interpret and communicate multi-parameter dynamic biological data.

Here, we describe the features and implementation of NetworkPainter. We present a mass cytometry timecourse of the human peripheral blood mononuclear cell (PBMC) immune signaling network [10] as an example use case. We conclude by discussing our future plans for NetworkPainter.

## Features

NetworkPainter provides researchers a web-based graphical interface to visually analyze cytometry data. NetworkPainter only requires a web browser with Adobe Flash Player. Below we briefly describe how to use NetworkPainter. Additional file 1 provides further information about how to use the software.

**Figure 1:**
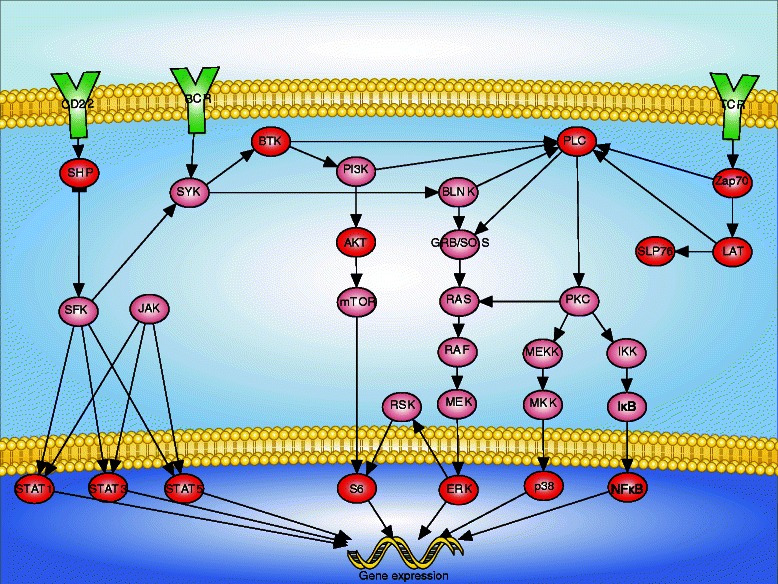
NetworkPainter enables researchers to quickly draw publication quality signaling diagrams. PBMC immune signaling diagram created with NetworkPainter. Diagram adapted from Bodenmiller et al., 2012 Figure S6 [10]. Observed and unobserved signaling nodes are colored dark and light red, respectively; extracellular receptors are colored green.

First, researchers use the graphical interface to draw a pathway diagram, formalizing their prior biological knowledge about their pathway, including its molecular components and their interactions. Users simply drag and drop molecules to add them to the diagram and assign them to subcellular compartments. Users can draw arrows by selecting two molecules and selecting “Draw arrow” from the right-click context menu. Users have full control over the graphical appearance of each molecule and compartment including its color, shape, and size. Twenty-six shapes are available including polygons, as well as several commonly used graphical representations of DNA, RNA, and protein. Figure 1 depicts a PBMC immune signaling diagram created with NetworkPainter.

To help users create visually pleasing diagrams, NetworkPainter provides an automatic layout tool to improve the arrangement and spacing of molecules in diagrams. In addition, users can import diagrams from the KEGG PATHWAY database [33] or from diagrams published by other users. Furthermore, NetworkPainter provides basic editing features including cut, copy, paste, undo, redo, auto save, find, zoom, and pan.

Second, users upload and annotate experimental data. Cytobank users use Cytobank’s existing graphical interface to create an experiment, upload flow or mass cytometry data, and annotate the individual, cell type, condition, dosage, channel, and timepoint of each measurement. Standalone users can upload data using the JSON format described in the online help.

Third, users link diagram nodes with experimental measurements and perturbations. Researchers use drop-down lists to select the experimental channel corresponding to each observed signaling molecule. Optionally, researchers can also use drop-down lists to enter the enhansive or repressive effect of each experimental perturbation.

Fourth, researchers can specify how to display the experimental individual, cell type, condition, and dosage dimensions of their data. Researchers can select two dimensions to display in small heatmaps below each experimentally linked signaling molecule. These heatmaps display the measurement of the corresponding experimental channel for each value of the two selected heatmap dimensions at each timepoint, averaged over the unselected heatmap dimensions. Optionally, NetworkPainter can cluster and order the selected heatmap dimensions using hierarchical agglomerative clustering [34] and optimal leaf ordering [35]. A legend and tooltips indicate the data plotted in each heatmap cell. These heatmaps are designed to help researchers visualize more of their data simultaneously, as well as compare measurements across individuals, cell types, conditions, and dosages.

Fifth, researchers can specify the averaging algorithm NetworkPainter uses to collapse the experimental data displayed in the signal molecules and heatmaps. Users can select among all of the statistics (e.g. mean, median, min, max), equations (e.g. raw, log, fold, arcsinh, difference), and controls (e.g. first row, first column, table min, table max) available in Cytobank’s illustrations. Researchers can also select the color mapping between the experimentally observed values and painted colors. The online help lists all of the available statistics, equations, controls, and colormaps.

**Figure 2:**
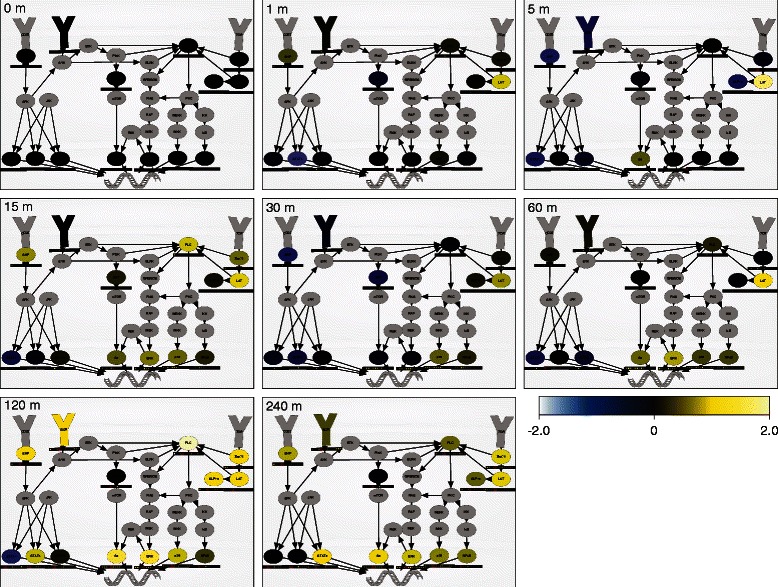
NetworkPainter visualizes multi-parameter data in the context of biological pathways. PBMC pathway painted with a time course of mass cytometry measurements obtained at 0, 1, 5, 15, 30, 60, 120, and 240 min post-LPS induction [10]. Animated pathway diagram highlights the differential cell type responses observed by Bodenmiller et al. Heatmaps indicate each node's median activity across the fourteen observed cell types (first row: CD14- monocytes; second row: CD14+ monocytes; third row: dendritic cells, IgM+ B cells, IgM- B cells, NK cells; last row: CD8+ T cells, CD4+ T cells). Nodes are colored by their mean value across all fourteen cell types. Yellow color indicates high activity; blue indicates low activity. An interactive, animated version of Figure 2 is available at http://covert.stanford.edu/networkpainter/KarrEtAl2014Fig2.

Sixth, researchers can use the playback controls at the bottom-left of the diagram to dynamically paint each experimentally perturbed or observed signaling molecule over the measured timecourse. After clicking the play button, NetworkPainter colors each experimentally linked signaling molecule according to the measured value of the corresponding experimental channel at the current timepoint and the selected colormap, averaged over all other experimental dimensions. NetworkPainter interpolates timepoints to produce smooth animations. Unobserved nodes are colored grey and optionally, the compartments and membranes are colored grey to emphasize the dynamics of the observed nodes. Optionally, small heatmaps below the signaling molecules enumerate the values of the experimental channel across the two selected heatmap dimensions. Users can also control the playback speed and looping. Figure 2 depicts eight static snapshots of a human PBMC immune signaling diagram painted by NetworkPainter with the first reported mass cytometry timecourse containing fourteen measured signaling nodes [10]. An interactive, animated version of Figure 2 is available at http://covert.stanford.edu/networkpainter/KarrEtAl2014Fig2. The case study below describes how NetworkPainter can be used to analyze this data.

Seventh, researchers can save diagrams to either the Cytobank or standalone server for later use and/or sharing with collaborators. Cytobank users can give collaborators permission to view or edit diagrams by granting permission to their associated experiments. Standalone users can use a simple graphical interface to grant read, write, or administer privileges to other users. Once collaborators are granted permission, they can edit a diagram. However, each diagram can only be edited by a single user at a time. Both Cytobank and standalone users can also publish diagrams to share them with all users.

Lastly, researchers can export diagrams to several image (GIF, JPEG, PNG, SVG) and animation (GIF, SWF) formats for use in papers, presentations, and websites. NetworkPainter also exports diagrams to the Biological Pathway Exchange (BioPax), CellML, and Systems Biology Markup Language (SBML) standards for use with other network analysis software programs, as well as to a JSON format which can be subsequently imported back into NetworkPainter. In addition, NetworkPainter can generate MATLAB scripts for Boolean simulations.

## Implementation

NetworkPainter was implemented as a web-based software program to enable platform independence, no installation, collaboration, and cloud-based computation. NetworkPainter is composed of a web-based graphical user interface, and a back-end server. The graphical interface provides users a diagram editor, paints signaling molecules with experimental data, and exports diagrams. The back-end server stores diagrams for later use, coordinates diagram permissions, helps export animated diagrams, and helps import diagrams from pathway databases.

The user interface was implemented using Adobe Flex [http://www.adobe.com/products/flex.html] using the Degrafa declarative graphics framework [http://code.google.com/p/degrafa]. Animation was implemented using Tweener [http://code.google.com/p/tweener]; automatic graph layout was implemented using GraphViz [36]; graphical export was implemented using Inkscape [http://www.inkscape.org]; and animation export was implemented using Adobe Flex.

The Cytobank server was implemented using JRuby [http://jruby.org], MySQL [http://www.mysql.com], Apache [http://httpd.apache.org], and Tomcat [http://tomcat.apache.org]. The standalone server was implemented using PHP [http://www.php.net], MySQL, and Apache. The servers and user interfaces communicate using JSON.

The NetworkPainter source code is freely available at http://github.com/CovertLab/NetworkPainter, including all of the code for the user interface and all of the code for the standalone server.

## Case Study: mass cytometry cell signaling timecourse

We illustrate the functionality of NetworkPainter using the first reported mass cytometry timecourse of human peripheral blood mononuclear cell (PBMC) signaling [10]. PBMCs are a diverse population of cells with distinct functions including B cells, T cells, NK cells, and macrophages. They are critical to both the innate and adaptive immune systems, helping the body identify and fight pathogens including viruses and bacteria.

Bodenmiller et al. used mass cytometry to investigate how PBMC subpopulations differentially respond to stimuli and drugs. They measured the effects of twelve stimuli on fourteen signaling nodes and ten surface markers in fourteen PBMC cell types at eight timepoints. In total, Bodenmiller et al. collected data from over 2,000 conditions.

Using SPADE, Bodenmiller et al. found complex dynamics, as well as significant cell type variability in the phosphorylation responses of the fourteen observed signaling nodes. In particular, they found that LPS rapidly induces p38, ERK, and NF-κB within 15–30 min in monocytes, followed by slower S6 induction at 2 h. In contrast, they also found that LPS slowly induces STAT3, STAT5, and ITK after 2 h in T cells and NK cells, and STAT1 in B cells after 4 h. Bodenmiller et al. attributed these differences in physiology to monocyte IL-6 secretion. Bodenmiller concluded that mass cytometry can be used to rapidly screen drug candidates against multiple cell types.

We used NetworkPainter to reexamine the Bodenmiller et al. dataset. First, we used NetworkPainter to draw a signaling diagram containing the fourteen observed signaling molecules (Figure 1). Next, we linked the diagram nodes to the observed channels, and used NetworkPainter to color the diagram with mass cytometry measurements (Figure 2). This enabled us to quickly contextualize, browse, and analyze the Bodenmiller et al. dataset. The animated diagram illustrated the same temporal signaling profile and cell type differences among monocytes, B cells, T cells, and NK cells reported by Bodenmiller et al. Furthermore, NeworkPainter's simple graphical interface allowed us to explore alternate network architectures and explanations for the observed dynamics and cell type variability. This example highlights the utility of NetworkPainter to help researchers quickly discern patterns from complex cytometry data.

## Results and Discussion

NetworkPainter is a web-based program for visualizing dynamic cytometry data in the context of animated pathway diagrams. NetworkPainter provides a simple graphical interface for drawing and painting pathway diagrams with experimental cytometry data either stored in the Cytobank platform or uploaded to the standalone version. NetworkPainter stores diagrams on its server to enable users to easily share diagrams with collaborators. NetworkPainter exports diagrams to a variety of image and animation formats, enabling users to create high-quality graphics for papers, presentations, and websites.

Going forward, we hope to integrate NetworkPainter with additional pathway and experimental databases to make it easier for researchers to analyze experiments with existing pathways without any complicated file conversion or importing. We also plan to make NetworkPainter compatible with mobile devices by implementing it using only JavaScript and HTML.

Overall, high-throughput technologies such as mass cytometry have the potential to provide valuable insights into the molecular circuits that govern biological behavior and disease. Data visualization software is needed to help researchers explore and analyze the large data sets created by these technologies. We believe that NetworkPainter is a valuable tool for analyzing multi-parameter dynamical data. Furthermore, we believe that NetworkPainter is a valuable communication tool. NetworkPainter can help researchers communicate complex data to the broader scientific community in a simple, intuitive graphical format.

## Availability and requirements

**Project name:** NetworkPainter

**Project home page:** Cytobank version: http://www.cytobank.org/networkpainter.html, standalone version: http://covert.stanford.edu/networkpainter

**Operating system(s):** Platform independent

**Programming language:** Actionscript, Ruby, PHP

**Other requirements:** Web browser, Flash player

**License:** Attribution Assurance License

**Any restrictions to use by non-academics:** None

NetworkPainter is available through the Cytobank cytometry platform at http://www.cytobank.org/networkpainter.html to analyze data stored in Cytobank. NetworkPainter is also freely available at http://covert.stanford.edu/networkpainter for use with any dynamic data. Source code is available at http://github.com/CovertLab/NetworkPainter under the Attribution Assurance License.

## Additional file

Additional file 1: NetworkPainter user instructions.

## Abbreviations

JSON: JavaScript object notation
PBMC: peripheral blood mononuclear cell
PHP: PHP: hypertext preprocessor
