# Supplementary material for: NetworkPainter: dynamic intracellular pathway animation in Cytobank
Source: BMC Bioinformatics. 2015 May 25;16:172. doi: 10.1186/s12859-015-0602-4 (PMC4491883; doi:10.1186/s12859-015-0602-4)
Supplement: Additional file 1: — NetworkPainter user instructions. [file 12859_2015_602_MOESM1_ESM.pdf]

Additional file 1. NetworkPainter user instructions

NetworkPainter-Cytobank

Requirements

The Cytobank version can be used with any web browser with the Adobe Flash Player. The Cytobank version does not require any installation.

Viewing demos diagrams

Please follow these steps to explore NetworkPainter using the demo diagrams:

1. Navigate to the NetworkPainter landing page at http://www.cytobank.org/networkpainter.html
2. Create a Cytobank account, and login
3. From the experiments inbox, select one of the select the experiments titled "NetworkPainter Demo – Human PBMC mass cytometry {*LPS, INF-α, ...*} timecourse (Bodenmiller et al., 2012)."
4. From the experiment's page, select "View Signaling Network Diagrams" in the left-hand side "actions" menu
5. Select a diagram
6. Use the playback controls at the bottom-left of the application to paint the diagram with data, and to pause the animation and control its speed

Getting started

Please follow these steps to draw and paint diagrams using the Cytobank version:

1. Navigate to http://www.cytobank.org/networkpainter.html
2. Create a Cytobank account, and login
3. Create an experiment, and upload data
4. From the experiment's detail page, click "View Signaling Network Diagrams" in the left-hand side menu
5. Click the "Create a New Diagram" button to create a new diagram
6. Use the graphical interface to draw a diagram
   (a) Use the toolbar to drag and drop new molecules onto the diagram, and follow the instructions in the pop-up windows:
      i. Choose a name and label
      ii. Select the corresponding experimental channel to link molecules with experimental data
      iii. Click "Add" to append the molecule to the diagram
   (b) Add edges by either
      i. Selecting two molecules (hold Ctrl and click two molecules), right-clicking, and then choosing "Draw arrow" from the context menu, or
      ii. Entering a Boolean rule into the "Regulation" field for each molecule
7. Use the playback controls at the bottom-left of the application to paint the diagram with data, and to pause the animation and control its speed

Detailed instructions and further help

Please see http://covert.stanford.edu/networkpainter for tutorials and further help or contact us at support@cytobank.org.

NetworkPainter-Standalone

Requirements

The standalone version can be used with any web browser with the Adobe Flash Player. The standalone version does not require any installation or user registration.

Viewing demo diagrams

Please follow these steps to explore NetworkPainter using the demo diagrams:

1. Navigate to http://covert.stanford.edu/networkpainter
2. Select the gallery link from the top menu
3. Choose a demo, and click its "View in NetworkPainter" link
4. Open NetworkPainter as a guest, or create a new account and login
5. Load an experiment
   (a) From the main menu select "Experiment" → "Manage experiments ..."
   (b) Highlight an experiment in the pop-up window, and click "Select" to paint the network with the experimental data
6. Control the animation using the playback controls at the bottom-left of the application

Getting started

Please follow these steps to draw and paint diagrams using the standalone version:

1. Navigate to http://covert.stanford.edu/networkpainter
2. Use the web form to either (a) register for an account and login which will allow you to privately save diagrams, or (b) use the software as a guest without any registration
3. Use the graphical interface to draw a diagram
   (a) Use the toolbar to drag and drop new molecules onto the diagram, and follow the instructions in the pop-up window:
      i. Choose a name and label
      ii. Click "Add" to append the molecule to the diagram
   (b) Add edges by either
      i. Selecting two molecules (hold Ctrl and click two molecules), right-clicking, and then choosing "Draw arrow" from the context menu, or
      ii. Entering a Boolean rule into the "Regulation" field for each molecule
4. Upload experimental data and link data to network molecules
   (a) From the main menu select "Experiment" → "Manage experiments ..."
   (b) Click "Add" in the pop-up window

(c) Select a JSON file containing experimental data (see the online help at http://covert.stanford.edu/networkpainter for documentation of the JSON format and an example)

(d) Use the "Channel-Biomolecule Associations" tab to select the experimental channel corresponding to each network biomolecule

(e) Highlight the experiment in the left side of the pop-up window, and click "Select" to paint the network with the uploaded data

5. Control the animation using the playback controls at the bottom-left of the application

Detailed instructions and further help

Please see http://covert.stanford.edu/networkpainter for tutorials and further help.
